# 1D NMR WaterLOGSY as an efficient method for fragment-based lead discovery

**DOI:** 10.1080/14756366.2019.1636235

**Published:** 2019-07-09

**Authors:** Claire Raingeval, Olivier Cala, Béatrice Brion, Marc Le Borgne, Roderick Eliot Hubbard, Isabelle Krimm

**Affiliations:** a Université de Lyon, CNRS, Université Claude Bernard Lyon 1, ENS Lyon, CRMN FRE 2034, Villeurbanne, France;; b Université de Lyon, Université Claude Bernard Lyon 1, Faculté de Pharmacie – ISPB, EA 4446 Bioactive Molecules and Medicinal Chemistry, SFR Santé Lyon-Est CNRS UMS3453 – INSERM US7, Lyon, France;; c YSBL, University of York, Heslington, York, UK;; d Vernalis (R&D) Ltd, Granta Park, Abington, Cambridge, UK

**Keywords:** WaterLOGSY, solvent-exposed, saturation transfer difference, binding mode, fragment-based lead discovery

## Abstract

WaterLOGSY is a sensitive ligand-observed NMR experiment for detection of interaction between a ligand and a protein and is now well-established as a screening technique for fragment-based lead discovery. Here we develop and assess a protocol to derive ligand epitope mapping from WaterLOGSY data and demonstrate its general applicability in studies of fragment-sized ligands binding to six different proteins (glycogen phosphorylase, protein peroxiredoxin 5, Bcl-x_L_, Mcl-1, HSP90, and human serum albumin). We compare the WaterLOGSY results to those obtained from the more widely used saturation transfer difference experiments and to the 3D structures of the complexes when available. In addition, we evaluate the impact of ligand labile protons on the WaterLOGSY data. Our results demonstrate that the WaterLOGSY experiment can be used as an additional confirmation of the binding mode of a ligand to a protein.

## 1. Introduction

Fragment-based lead discovery (FBLD)^1^^,^^2^ is maturing as an effective method for generating small molecules that modulate the activity of biological molecules, usually proteins. The central idea is to screen a small number (typically 1000 s) of low molecular weight (usually less than 250 Da) compounds against a protein target of interest, mostly using a sensitive biophysical technique that can detect binding with an affinity as weak as low mM (K_D_ for dissociation)^2–4^. Hit fragments are then optimised by either growing or merging features of compounds to generate lead molecules. Such molecules can subsequently be used as tools to probe the biology of a protein or be further optimised to give clinical candidates such as the recently approved medicines, vemurafenib, and venetoclax^5^^,^^6^. While it is relatively straightforward to find many (100 s of) fragments that bind for most proteins^7^, it may be more difficult to characterise the molecular interactions within the protein-fragment complexes when X-ray crystallography is not successful. Yet the assessment of the ligand binding mode is a critical information that provides important guidance for the identification of suitable modification locations in fragment optimisation.

NMR is a well-established method for screening fragment libraries^8^. One of the most popular ligand-observed NMR experiments is the saturation-transfer difference (STD) experiment^9^^–^^12^. The STD spectrum displays the NMR peaks of a small molecule only if the latter binds to the protein in the timescale of the experiment. In addition, the size of the individual ^1^H STD signals reflects the proximity of the corresponding proton to the protein. We have previously shown that fragment binding mode can be characterised using the so-called epitope mapping approach, through the quantitative analysis of STD spectra^13^. The WaterLOGSY experiment^14^^,^^15^ is another popular experiment for the studies of molecular interactions^16^. WaterLOGSY is used for the identification of protein ligands and the determination of protein-ligand binding constants^17^. The experiment provides some key advantages. First, compound solubility can be checked which prevents the selection of false positive hits due to compound aggregation in the fragment screening experiments^14^^,^^15^. Also, WaterLOGSY has been reported to be more sensitive compared to STD^18^^–^^20^, and the development of dissolution dynamic nuclear polarisation (D-DNP) WaterLOGSY may provide an even more sensitive NMR method to detect protein-ligand interactions^21^. Finally, WaterLOGSY is of particular interest for low proton density receptors such as nucleic acids^22^ or for highly solvated proteins such as amyloid fibers^23^.

The WaterLOGSY experiment involves several mechanisms for the magnetisation transfer from the water to the protein and then to the ligand^11^^,^^14^^,^^15^^,^^24^^,^^25^: (1) direct transfer from water molecules immobilised in the protein binding site; (2) chemical exchange between water and protein labile protons (amide, hydroxyl, amino, etc.) (3) transfer from the water molecules found in the protein surface via the protein-ligand complex. Thus, the solvent accessibility of ligands bound to proteins can be assessed using WaterLOGSY. The first demonstration of the use of the WaterLOGSY experiment to measure bound-ligand solvent accessibility was published in 2008 by the group of Günther^24^^,^^25^, with the quinone oxidoreductase 2 (NQO2) and two dehydrogenases as protein targets. Since then, the group of Konrat published in 2017 a new approach to study the ligand solvent accessibility and proposed to analyse WaterLOGSY-based titration performed on a perdeuterated protein sample^26^. The approach offers the advantage of being applicable to low-molecular-weight proteins such as bromodomains. However, a specific protein preparation is required, which can be costly.

Here, we report an extensive study for the assessment of the binding mode of small organic compounds bound to proteins, through the WaterLOGSY experiment. For the first time, we investigate the impact of ligand exchangeable protons on the WaterLOGSY analysis. We compare, for six proteins with molecular weights ranging from 22 to 180 kDa, the WaterLOGSY spectra to the STD spectra recorded for each protein-ligand complex. Proteins are the protein peroxiredoxin 5 (PRDX5), B-cell lymphoma-extra large (Bcl-x_L_), Myeloid cell leukaemia 1 protein (Mcl-1), human serum albumin (HSA), HSP90, and glycogen phosphorylase (GP).

## 2. Materials and methods

### 2.1. Biology

Glycogen phosphorylase from a rabbit muscle and human serum albumin fatty acid free were purchased from Sigma (the references are P1261 and A3782, respectively). Protein peroxiredoxin 5, Bcl-x_L_, Mcl-1, and the N-terminal domain (9–236) of HSP-90 were produced and purified at IBCP-Lyon “Bioengineering of proteins” facility using *Escherichia coli* as previously described^27^^,^^28^. The following buffers were used: peroxiredoxin: 5 pH 7.4, NaCl 137 mM, KCl 2.8 mM, Na_2_HPO_4_ 10 mM, KH_2_PO_4_ 1.8 mM, and 3 mM DTT; HSP90: Tris-HCl 20 mM pH 7.5 NaCl 100 mM and 1 mM EDTA; Bcl-x_L_: pH 7, Na_2_HPO_4_ 25 mM and 3 mM DTT; Mcl-1: pH 8, Na_2_HPO_4_ 50 mM, NaCl 70 mM, and 1 mM EDTA.

### 2.2. NMR experiments

NMR samples for STD experiments consisted of 400 µM fragment in 0.5% DMSO-d_6_ and 10% D_2_O (v/v) together with a protein concentration of 20 µM for peroxiredoxin and HSP90, 10 µM for Bcl-x_L_, 15 µM for Mcl-1, 2 µM for glycogen phosphorylase, and 5 µM for HSA.

Standard 1 D, STD, and WaterLOGSY NMR spectra were acquired at 20 °C with an Agilent Inova 600 MHz NMR spectrometer, equipped with a room temperature 5 mm triple-resonance inverse probe with z-axis field gradient and a Bruker 600 MHz NMR spectrometer, equipped with a Z-gradient cryoprobe.

WaterLOGSY mixing time was 1.5 s, and additional experiments were performed with 0.75 s and 0.25 s mixing time. Both STD and WaterLOGSY spectra were recorded for each sample. WaterLOGSY spectra in the absence of protein receptor were recorded with 400 µM compound. All the NMR experiments were performed at 293 K with excitation sculpting to suppress peaks from water. 1 D and STD experiments were performed using identical experimental conditions (spin lock, interscan delays), and parameters for the STD experiments (saturation frequency and saturation time) were identical for all samples. Selective saturation of the protein NMR spectrum was achieved with the decoupler offset 2500 Hz upfield from the carrier frequency, and non-saturation control was performed at 15,000 Hz downfield. Saturation time was set to 2 s for all experiments. The STD factors were calculated according to the following equation: *R*=(I_STD_/I_1D_)×100) were I_STD_ and I_1D_ are the intensity of proton resonances of the STD and the 1 D proton spectra, respectively. STD spectra were normalised by setting the largest observed signal to 100% of the equivalent 1 D signal.

### 2.3. Molecular docking

All docking computations were performed with AutoDock4.2.3^29^. Two-hundred independent runs were conducted for each fragment using the Genetic Algorithm with standard settings. Structure PDB entry 3MNG containing PRDX5 in interaction with DTT and 1AO6 containing HSA were used as the 3 D template for the docking. Protein and fragment structures were prepared with AutoDockTools (ADT). The standard AutoDock-potential scoring function was used as previously explained^27^.

## 3. Results and discussion

### 3.1. Quantitative comparison of WaterLOGSY signals of ligands free and bound to the protein receptor

In the WaterLOGSY experiment, the molecules that interact with water via water-ligand-protein or protein-ligand complexes exhibit a negative NOE with water (positive WaterLOGSY signal), while the molecules that do not bind the protein have weak and positive intermolecular NOEs with water (negative WaterLOGSY signal). Although the overall change of sign is dependent on the ligand/protein ratio, binding and non-binding ligands can be discriminated in a WaterLOGSY spectrum, as they display opposite signs for their corresponding peaks^14^^,^^15^.

In the so-called SALMON effect, the sign of the WaterLOGSY peaks was observed to remain negative (as for free ligands) for solvent-exposed protons of the 5-(aziridin-1-yl)-2,4-dinitrobenzamide bound to NQO2, compared to the peaks from buried protons of the ligand. For cases where no proton displayed negative WaterLOGSY peaks, the intensities of individual protons were compared with those in the 1 D spectrum^24^^,^^25^.

**Figure 1.**
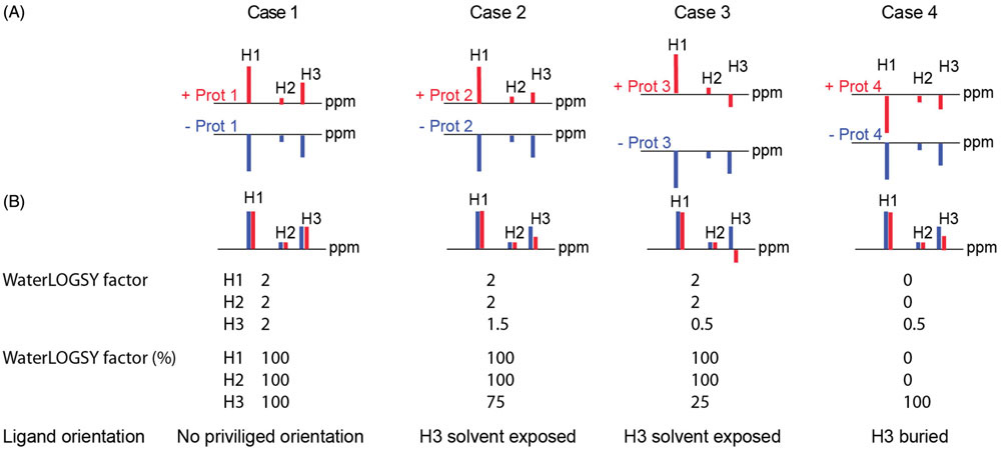
Schematic representation of WaterLOGSY-based characterisation of protein ligands. Four different cases are illustrated for a compound exhibiting three protons H1, H2, and H3 (see the text). (A) Theoretical WaterLOGSY spectra, in the absence (blue) and in the presence (red) of the protein receptor, are displayed. To facilitate the comparison of the relative intensities, the WaterLOGSY ones are normalised, using the peak with the strongest WaterLOGSY intensity in the absence of protein as the reference peak (here H1). (B) Theoretical WaterLOGSY spectra, in the absence (blue) and in the presence (red) or the protein receptor, with the WaterLOGSY spectrum of the free compound, phased to allow the superimposition with the WaterLOGSY spectrum of the bound compound. The WaterLOGSY factors are calculated using hypothetical intensities, using the equation |(I_WLOGSY+_) – (I_WLOGSY−_)|/(I_WLOGSY−_)| and reported for the three protons H1, H2, and H3. The values are then normalised, similarly to the calculation of STD factors, with the highest value set to 100%. Calculations for the protons (H1, H2 and H3) are detailed here. Case 1 ((I_WLOGSY+_) = − (I_WLOGSY−_) for H1, H2 and H3): WLOGSY_H1_ = WLOGSY_H2_ = WLOGSY_H3_ = 2 and are all normalised to 100%. Case 2 ((I_WLOGSY+_) = − (I_WLOGSY−_) for H1 and H2; (I_WLOGSY+_) = − 1/2 (I_WLOGSY−_) for H3): WLOGSY_H1_ = WLOGSY_H2_ = 2 and WLOGSY_H3_ = 1.5, corresponding to 100%, 100%, and 75%, respectively. Case 3 ((I_WLOGSY+_) = − (I_WLOGSY−_) for H1 and H2; (I_WLOGSY+_) = 1/2 (I_WLOGSY−_) for H3): WLOGSY_H1_ = WLOGSY_H2_ = 2 and WLOGSY_H3_ = 0.5, corresponding to 100%, 100%, and 25%, respectively. Case 4 ((I_WLOGSY+_) = (I_WLOGSY−_) for H1 and H2; (I_WLOGSY+_) = 1/2 (I_WLOGSY−_) for H3): WLOGSY_H1_ = WLOGSY_H2_ = 0 and WLOGSY_H3_ = 0.5, corresponding to 0%, 0%, and 100%, respectively.

Here we propose to compare the WaterLOGSY intensities of the bound ligand to the WaterLOGSY intensities of the free compound, to highlight changes in intensities upon protein binding, as the WaterLOGSY intensities of protons in organic compounds do not necessarily reflect the intensities observed in normal 1 D spectra. This is due to the WaterLOGSY mechanism that involves magnetisation transfer from bulk water. Therefore, we propose the following protocol to quantitatively evaluate the WaterLOGSY signals of the protons of a compound bound to its receptor. We define a WLOGSY factor calculated using magnitudes of WaterLOGSY signals as: WLOGSY factor=|(I_WLOGSY_+) – (I_WLOGSY_−)|/|(I_WLOGSY_−)|, with IWLOGSY+ and IWLOGSY−  the WaterLOGSY peak intensities in the presence and absence of the protein. The values are then normalised with the highest value for the WLOGSY factor of the corresponding proton set to 100%. The smallest WLOGSY factor value corresponds to the most solvent-exposed proton. The different possible scenarios are illustrated schematically in Figure 1, where theoretical WaterLOGSY spectra are displayed for a compound containing three protons (Figure 1(A)). For clarity, the intensities are normalised for the spectra in the presence or absence of the protein, using the proton H1 (exhibiting the strongest WaterLOGSY intensity in the absence of the protein) as a reference. In Figure 1(B), the spectra are phased to allow superimposition. In this way, the comparison of the two WaterLOGSY spectra is similar to the comparison of STD and STDoff spectra, with the WaterLOGSY bound spectrum corresponding to the STD spectrum and the WaterLOGSY free spectrum corresponding to the STDoff spectrum^30^. In cases 1–4, the proton H1 displays a change of WaterLOGSY signal intensity between the free and bound WaterLOGSY spectra.

**Figure 2.**
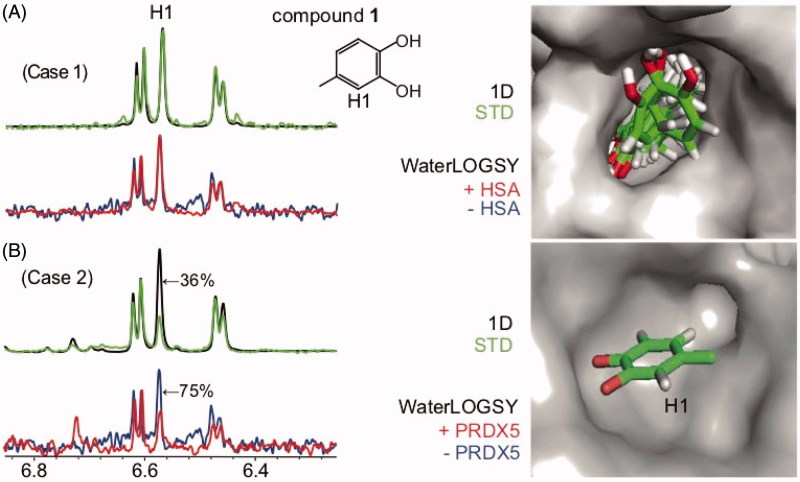
WaterLOGSY spectra for a compound bound to a protein target (A) without any defined orientation or (B) through a defined orientation (cases 1 and 2): (A) Compound **1** bound to HSA (K_D_ > 1 mM) B) Compound **1** bound to PRDX5 (K_D_=330 μM). The STD spectrum is shown in green, with the STD_off_ spectrum (1 D) superimposed in black. The WaterLOGSY spectrum in the absence of the protein is shown in blue, the WaterLOGSY spectrum in the presence of the protein is shown in red. The solvent-exposed proton H1 identified using WaterLOGSY and STD experiments is labelled. The corresponding WaterLOGSY and STD factors are indicated. The X-ray structure is shown for compound **1** bound to PRDX5 (4K7N) and compound **1** docked in HSA. All docking computations were performed with AutoDock4.2.3^29^.

**Figure 3.**
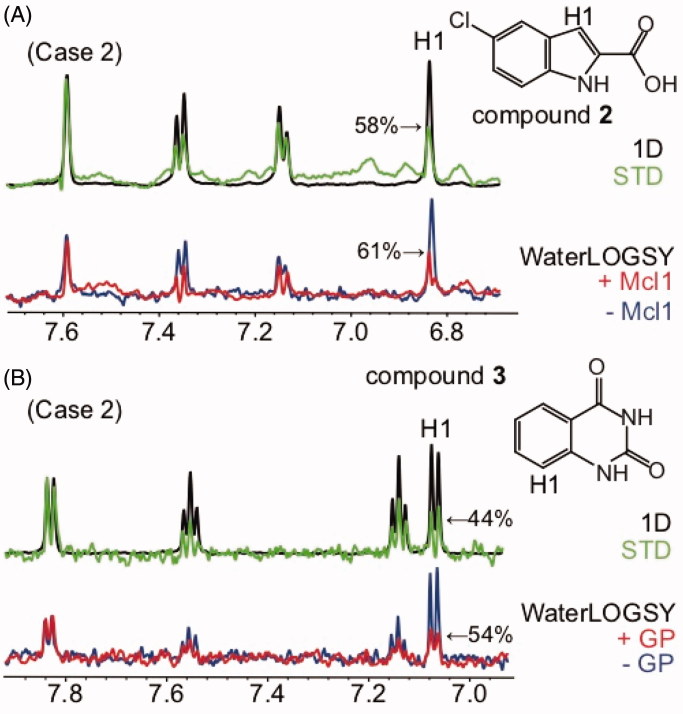
WaterLOGSY detects that fragments bind the protein target through a defined orientation and identifies solvent-exposed ligand protons: case 2. (A) Compound **2** bound to Mcl-1 (K_D_ not determined) (B) Compound **3** bound to GP (K_D_ >1 mM). The STD spectrum is shown in green, with the STD_off_ spectrum (1 D) superimposed in black. The WaterLOGSY spectrum in the absence of the protein is shown in blue, the WaterLOGSY spectrum in the presence of the protein is shown in red. The solvent-exposed proton H1 identified using WaterLOGSY and STD experiments is labelled. The corresponding WaterLOGSY and STD factors are indicated.

**Figure 4.**
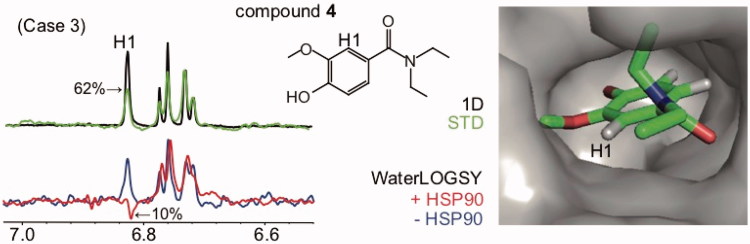
WaterLOGSY detects that fragments bind the protein target through a defined orientation and identifies solvent-exposed ligand protons: case 3 (both positive and negative WaterLOGSY peaks are observed). Compound **4** binds to HSP90 (K_D_=790 μM). The STD spectrum is shown in green, with the STD_off_ spectrum (1 D) superimposed in black. The WaterLOGSY spectrum in the absence of the protein is shown in blue, the WaterLOGSY spectrum in the presence of the protein is shown in red. The solvent-exposed proton H1 identified using WaterLOGSY and STD experiments is labelled. The corresponding WaterLOGSY and STD factors are indicated. The X-ray structure of the complex is shown (2XDL).

In case 1 A, the WaterLOGSY signals display opposite signs upon binding to the protein. However, when the WaterLOGSY spectrum of the free compound is phased to allow superimposition with the WaterLOGSY spectrum of the bound compound, the WaterLOGSY intensities are similar for each of the three protons (case 1). As a consequence, the WLOGSY factors are 100% for the three protons, indicating that the binding has no impact on the WaterLOGSY intensities. This arises either because the ligand binds to the protein in multiple orientations in one or more binding sites, or because the ligand is fully buried in the protein, or because no specific proton is solvent-exposed in the corresponding binding mode (see Figure 2(A)). In case 2, the WaterLOGSY signals exhibit opposite signs upon binding to the protein but the *relative* WaterLOGSY intensity is weaker for proton H3 (case 2, see Figure 2(B) and Figure 3). The WLOGSY factor is 75% for proton H3, indicating that proton H3 is solvent-exposed in the complex structure. In case 3, the WaterLOGSY signal of proton H3 still displays a negative sign, while protons H1 and H2 display positive WaterLOGSY signals upon binding to the protein (case 3). This case is experimentally observed for compounds bound to low molecular weight receptors (25–30 kDa), due to the reduced spin diffusion (see Figure 4). The WaterLOGSY mechanisms involving the cross-relaxation rates between the proton and proximal protein protons and the cross-relaxation rates between the proton and water molecules are in competition, and the relative influence of these mechanisms depends on the receptor molecule weight. In case 3, the WLOGSY factor is 25% for proton H3, indicating that proton H3 is solvent-exposed in the complex structure. Finally, in case 4, the WaterLOGSY signals of protons H1, H2, and H3 still display a negative sign, but proton H3 exhibits a weaker WaterLOGSY signal intensity upon protein binding. This implies that proton H3 is less solvent-exposed than protons H1 and H2. Below, each of these 4 cases is illustrated with experiments on six different protein receptors.

### 3.2. Structural information from WaterLOGSY spectra

We first demonstrate that WaterLOGSY is sensitive to the orientation of the bound ligand and can discriminate specific (one defined orientation, case 2) from non-specific binding (multiple orientations, case 1). Figure 2 shows the WaterLOGSY spectra of compound **1** (4-methylcatechol) free and bound to PRDX5 (36 kDa) and HSA. As highlighted in Figure 2(B), the proton H1 of compound **1** has a WLOGSY factor of 75%, consistent with its solvent exposure in the crystallographic complex structure and previously reported STD-NMR data^27^. By contrast, as can be seen in Figure 2(A), all the protons of compound **1** bound to HSA exhibit WLOGSY factors of 100%. This shows that compound **1** binds HSA without any preferred orientation, in agreement with the propensity of the protein to bind a large diversity of compounds nonspecifically^31^.

**Figure 5.**
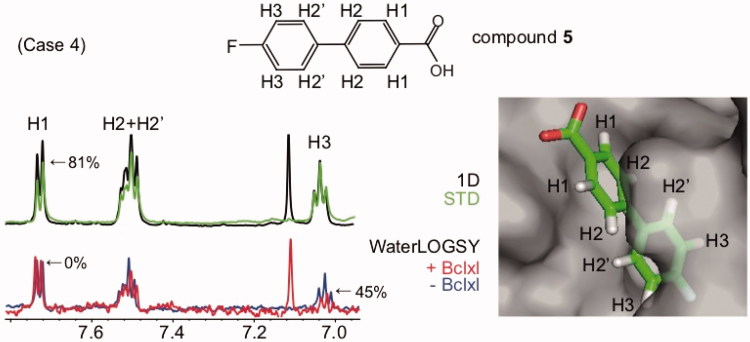
WaterLOGSY detects that fragments bind the protein target through a defined orientation and identifies solvent-exposed ligand protons: case 4 (all WaterLOGSY peaks display the same sign for the bound and free compound). Compound **5** binds to the Bcl-xL protein (K_D_=300 μM). The STD spectrum is shown in green, with the STD_off_ spectrum (1 D) superimposed in black. The WaterLOGSY spectrum in the absence of the protein is shown in blue, the WaterLOGSY spectrum in the presence of the protein is shown in red. Due to the low molecular weight of the protein, the sign of the WaterLOGSY is the same for the free and bound compound (as shown in Figure 1, case 4) and therefore the two WaterLOGSY spectra are not 180° out of phase in this particular case. Therefore, in this case, the solvent-exposed protons exhibit similar relative intensities in the presence or absence of the protein (WaterLOGSY factors is zero). The solvent-exposed proton H1 identified using WaterLOGSY and STD experiments is labelled. The corresponding WaterLOGSY and STD factors are indicated. The peak at 7.1 ppm corresponds to one of the imidazole resonances. The NMR structure of the complex is shown (1YSG).

To further demonstrate that WaterLOGSY generates structural information, we report WaterLOGSY spectra for four other proteins, Mcl-1, and GP (Figure 3), the N-terminus of HSP90 (Figure 4), and Bcl-x_L_ (Figure 5). Mcl-1 and Bcl-x_L_, anti-apoptotic Bcl-2 family members frequently upregulated in cancer, are representatives of proteins involved in protein-protein interactions. GP is a representative of allosteric enzymes and has been chosen because of its high molecular weight (180 kDa). HSP90, a widely studied target in oncology and the subject of multiple fragment and structure-based discovery campaigns^32^, was selected for its low molecular weight (25 kDa) and for the well characterised X-ray structures of fragments binding to the protein.

Examples for Mcl-1 and GP binding to compound **2** 5-chloro-1*H*-indole-2-carboxylic acid and **3** 2,4-(1*H*,3*H*)-quinazolinedione, respectively, are shown in Figure 3. In both cases, the WaterLOGSY spectra identify solvent-exposed protons of the bound ligands, in complete agreement with the STD-based epitope mapping (Figure 3). Those are typical examples of case 2 displayed in Figure 1.

The theoretical case 3 shown in Figure 1 may be observed for protein targets with low molecular weights (below 25 kDa). Due to the low spin diffusion, a solvent-exposed proton can exhibit a negative WaterLOGSY peak. This case is illustrated for compound **4** (*N*,*N*-diethylvanillamide) bound to the N-terminal domain of HSP90 (Figure 4). The WaterLOGSY spectrum suggests that the proton H1 of compound **4** is solvent-exposed upon binding to HSP90 (WLOGSY factor is 10%). This observation is consistent with the STD data and the published X-ray structure (Figure 4)^32^.

Finally, case 4 is exemplified with Bcl-x_L_ (22 kDa) (Figure 5). If the ligand/protein ratio is large, in particular for low molecular weight receptors, the WaterLOGSY spectrum of the bound ligand may maintain a negative sign for all protons, as shown in Figure 1, case 4. This is not problematic for the analysis of the ligand orientation, as illustrated in Figure 5 where the WaterLOGSY spectra of compound **5** (4′-fluoro-[1,1′-biphenyl]-4-carboxylic acid) free and bound to Bcl-x_L_ are compared. While both WaterLOGSY spectra display the same sign, there are differences in the WaterLOGSY intensities. In this case, solvent-exposed protons display unchanged WaterLOGSY intensities (the corresponding protons are not influenced by the binding), while buried protons exhibit weak intensities. For compound **5** bound to Bcl-x_L_, STD spectra indicate that protons H1 are solvent-exposed, while protons H3 are buried in the protein surface. In agreement with the STD data, the relative WaterLOGSY signal intensity of protons H1 is not modified, suggesting that protons H1 are solvent-exposed. By contrast, the WaterLOGSY signal intensity of protons H3 is weaker upon binding, showing that protons H3 are buried in the protein-ligand complex. Those observations are in agreement with the NMR structures of the fragment-protein complex^33^.

Those six different examples show that the WaterLOGSY experiment can provide information on ligand binding mode for variously sized proteins, and demonstrates that it is not necessary to adjust (increase) the protein concentration in order to obtain positive WaterLOGSY signals (a change in the WaterLOGSY sign upon binding), since comparison of WaterLOGSY intensities for the protons of free and bound ligands provides the required information.

### 3.3. Influence of ligand exchangeable protons on the WaterLOGSY experiment

As described by Dalvit, the WaterLOGSY intensity depends, both for the free and bound ligands, on the presence of ligand exchangeable protons^14^^,^^15^. It is acknowledged that labile protons display positive exchange peaks in WaterLOGSY spectra even in the absence of the protein. This mechanism might, therefore, affect the WaterLOGSY intensities of the protons located nearby labile protons (distance <5 Å), due to the 1/R^6^ dependence of the NOE transfer. A proton located near a labile proton (such as OH and NH) should experience a large negative NOE. It might, therefore, be difficult to identify solvent-exposed ligand protons (through small WLOGSY factors) when the latter are nearby labile protons. This will depend on the balance between the different effects. The sensitivity of the WaterLOGSY experiment is mainly due to an efficient magnetisation transfer via spin diffusion through the protein complex. Consequently, the WaterLOGSY signal is larger for proteins with large correlation times. It is for this reason that WaterLOGSY experiments are typically achieved with long mixing times ranging from 1 s to 2 s. The signal-to-noise ratio rapidly decreases when shorter mixing times are used. By contrast, the magnetisation transfer involving labile protons of the ligand remains highly efficient at shorter mixing times.

**Figure 6.**
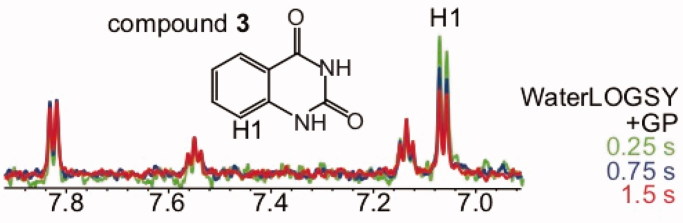
Influence of the mixing time on the WaterLOGSY intensities for protons positioned proximal to exchangeable protons. The WaterLOGSY spectra of compound **3** bound to GP (K_D_ >1 mM) with mixing times of 1.5 s, 0.75 s, and 0.25 s are shown in red, blue and green, respectively. The WaterLOGSY peak intensity of proton H1 increases when the mixing time decreases.

**Figure 7.**
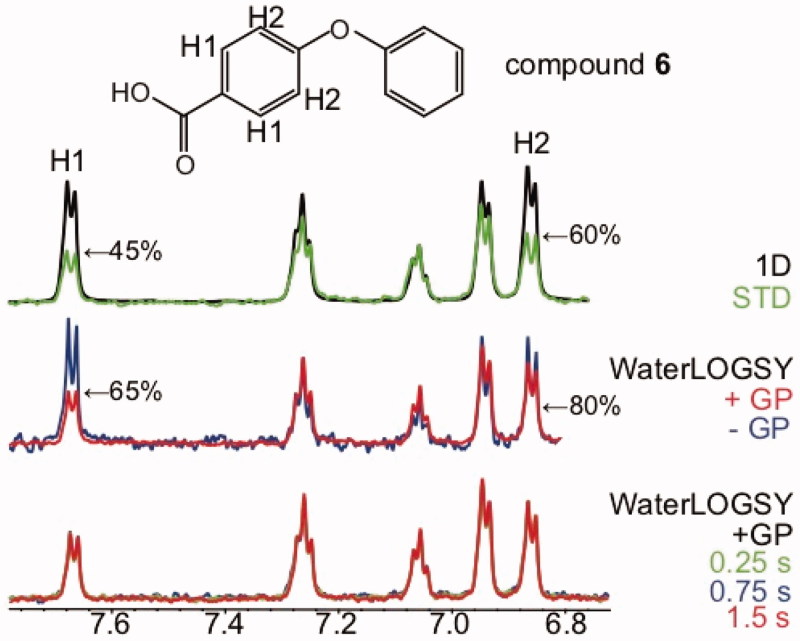
The mixing time has no impact on the WaterLOGSY intensities in the absence of proximal exchangeable protons. The WaterLOGSY spectra of compound **6** bound to GP (K_D_ >1 mM) with mixing times of 1.5 s, 0.75 s, and 0.25 s are shown in red, blue and green, respectively. The WaterLOGSY peak intensity of proton H1 increases when the mixing time decreases. The STD spectrum is shown in green, with the STD_off_ spectrum (1 D) superimposed in black. The WaterLOGSY spectra in the absence of the protein (blue), and in the presence of the protein (red) recorded with 1.5 s mixing time are displayed. The solvent-exposed protons H1 and H2 identified using WaterLOGSY and STD experiments are labelled. The corresponding WaterLOGSY and STD factors are indicated.

To study the influence of exchangeable protons, WaterLOGSY experiments were performed with mixing times of 1.5, 0.75, and 0.25 s for 20 protein-fragment complexes. We have compared the relative intensities of the WaterLOGSY peaks of the free and bound ligand states at the three different mixing times. As reported in Table S1, in the absence of exchangeable protons located nearby (distance <5 Å) the *relative* WaterLOGSY signal intensities of the ligand protons are not modified when the mixing time decreases. By contrast, for protons located *near* labile protons, the mixing time has an effect, demonstrating that exchangeable protons influence the intensities of the WaterLOGSY peaks for protons located nearby. One example is reported in Figure 6 for compound **3** (2,4-(1*H*,3*H*)-quinazolinedione) bound to GP. Using 1.5 s WaterLOGSY mixing time, proton H1 is shown to be solvent-accessible (see Figure 3(B)), displaying a weak WaterLOGSY intensity, in agreement with the STD spectrum. At lower mixing times, the peak intensity of proton H1 increases (Figure 6). Therefore, using a 0.25 s WaterLOGSY mixing time, proton H1 does not appear as solvent-accessible. This is due to the polarisation transfer with exchangeable protons, which dominates – at low mixing time – the polarisation transfer through the protein. In conclusion, these results indicate that the structural analysis should be performed with long mixing times (close to 1.5 s) to minimise the influence of exchangeable protons. As reported here for the protein-ligand complexes displayed in Figures 2, 3, 4, 5 and 7, as well as the 20 protein-fragment complexes reported in Table S1, the WaterLOGSY-based structural information is very similar to the STD-based structural information, when WaterLOGSY experiments are performed with long mixing times (1.5 s).

**Table 1.** Advantages and disadvantages of the STD and WaterLOGSY experiments for assessing solvent accessibility of ligands bound to receptors.

Description	STD	WaterLOGSY
Ligand	Artifacts are observed for methyl containing compounds, if the saturation frequency is not correctly chosen	No artifacts for methyl containing compounds
Receptor	Not sensitive for nucleic acids or low-molecular-weight receptors	Suitable for receptors such as nucleic acids; suitable for low-molecular-weight receptors (comparison of bound and free WaterLOGSY spectra)
Ligand/protein ratio	Can be performed for large ligand/protein ratios (1000:1)	The sensitivity of the experiment decreases for large ligand/protein ratios
Structural information	STD factors are sensitive to the T1 relaxation of the ligand protons; identification of ligand protons displaying weak solvent-accessibility (is particularly interesting for fragment screening); no artifacts due to exchangeable ligand protons	WaterLOGSY factors are sensitive to T1 relaxation of the ligand protons; WaterLOGSY factors are sensitive to the presence of exchangeable protons; structural information may be lost for protons exhibiting weak solvent-accessibility if they are located near exchangeable protons; recording several experiments using different mixing times can be useful

In the absence of exchangeable protons proximal to the protons under analysis, there is no impact of the WaterLOGSY mixing time on the structural information, as shown for example with compound **6** (4-phenoxybenzoic acid) bound to GP (Figure 7).

### 3.4. WaterLOGSY-based information compared to solvent accessibility: quantitative analysis

**Figure 8.**
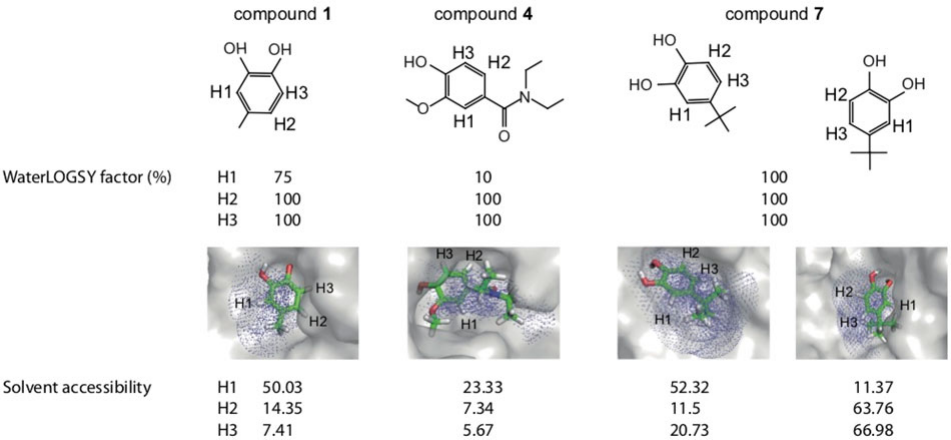
WaterLOGSY factors and solvent accessibility of protons for compound **1** bound to PRDX5 (K_D_=330 μM), compound **7** bound to PRDX5 (K_D_=50 μM), and compound **4** bound to HSP90 (K_D_=790 μM). The WaterLOGSY factors and the solvent accessibility calculated with Pymol are indicated for the different protons of the compounds.

The WaterLOGSY factors and the calculated solvent-accessibilities for compounds **1**, **4**, and **7** are shown in Figure 8. As expected, the protons exhibiting weaker WaterLOGSY factors correspond to the most solvent-exposed protons in the corresponding protein-ligand 3 D structures. For weak-affinity ligands such as fragment-like compounds, the comparison of WaterLOGSY factors between two analogues may be used to assess the impact of a chemical group on the binding mode of the ligand. This is illustrated in Figure 8 for compound **1** (4-methylcatechol, see also Figure 2) and compound **7** (4-tert-butylcatechol) bound to PRDX5. According to the WaterLOGSY factors, the binding mode compound **7** is modified in comparison to that of compound **1**. This observation is in agreement with the X-ray structure of the complex of compound **7** with PRDX5, in which two binding modes are observed (while only one binding mode was observed for compound **1**) (Figure 8). Depending on the binding modes, the proton H1 is either buried into the protein or solvent-exposed. The experimental WaterLOGSY factor is averaged, and no privileged orientation is observed. This result shows that WaterLOGSY-based structural information can be particularly useful to compare the binding properties of similar compounds.

### 3.5. Influence of the ligand affinity

The dissociation constant for fragment binding to proteins typically ranges from low micromolar to millimolar. In the examples reported here, the affinities are 330 µM for compound **1** bound to PRDX5^27^, 790 µM for compound **4** bound to HSP90^32^, greater than 1 mM for compound **3** and **6** bound to GP (unpublished data), 300 µM for compound **5** bound to Bcl-xL^33^ and 50 µM for compound **7** bound to PRDX5^27^. One feature of the examples for PRDX5 discussed here is that a weakly binding fragment (**1**, 330 µM) can bind with a defined orientation whereas a stronger binding compound (**7**, 50 µM) binds in two alternate orientations. These differences are seen in the X-ray crystal structures and are reported by the WaterLOGSY signals. This suggests that this WaterLOGSY-based method can be used to determine the binding orientation for fragments binding with a range of affinities to a protein target.

### 3.6. Assessing the epitope mapping of ligands using STD and WaterLOGSY

For assessing the epitope mapping of ligands bound to proteins, we strongly recommend recording both the STD and WaterLOGSY experiments. In most cases, this can be achieved using the same sample, even if the STD experiments require less protein quantity than the WaterLOGSY experiments. Advantages and disadvantages of the STD and WaterLOGSY experiments are reported in Table 1. In case of a disagreement between STD and WaterLOGSY, we suggest recording WaterLOGSY experiments at long and low mixing times, as shown in Figure 6, to better evaluate the impact of exchangeable ligand protons (typically when a ligand proton is identified as solvent-exposed in STD but not in WaterLOGSY). The opposite case, when a WaterLOGSY signal is observed while it is not observed in an STD experiment, might indicate the presence of a bound water molecule^34^. As recently reported, the presence of water molecules in the protein-ligand complex can also be assessed through the analysis of WaterLOGSY experiments performed on deuterated protein samples^26^.

## 4. Conclusion

Our results clearly demonstrate that the comparison of WaterLOGSY intensities for a small molecule in its free and protein-bound forms allows the identification of the ligand solvent-exposed protons. We have reported experimental data for the four scenarios that can be observed in the WaterLOGSY experiments, together with the structural information that can be inferred from these experiments. More importantly, we have used six different protein targets ranging from 22 to 180 kDa to establish the large applicability of the approach. For three of the examples studied here (Bcl-x_L_, PRDX5, and HSP90), published descriptions of compound optimisation confirm that these solvent-exposed positions on the fragment are used in compounds that bind with high affinity. One key point is the investigation of the influence of ligand exchangeable protons. As shown, two WaterLOGSY mixing times should be used to avoid mistakes in the assessment of proton solvent accessibility.

The approach does not require any structural information for the protein. Used alongside STD measurements, the WaterLOGSY protocols described here provide additional confidence in the binding mode for ligands binding to a protein. Such confidence is important for molecular design in the absence of a complete structure determination to guide the medicinal chemist in the optimisation of fragments, a crucial step in fragment-based drug discovery. The approach should be particularly valuable for cases where a fragment binds with a defined orientation but structural information (derived from X-ray crystallography or protein-observed NMR spectroscopy) is not available.

## Supplementary Material

Supplemental Material

## References

[1] Erlanson DA, Fesik SW, Hubbard RE, et al. Twenty years on: the impact of fragments on drug discovery. Nature Rev Drug Discov 2016;15:605–19. 27417849 10.1038/nrd.2016.109

[2] Erlanson DA Introduction to fragment-based drug discovery and X-ray crystallography In: Davies T, Hyvönen M, eds. Fragment-based drug discovery and X-ray crystallography. Topics in current chemistry. Vol. 317 Berlin and Heidelberg: Springer; 2012:1–32. 10.1007/128_2011_180 21695633

[3] Silvestre HL, Blundell TL, Abell C, Ciulli A Integrated biophysical approach to fragment screening and validation for fragment-based lead discovery. Proc Natl Acad Sci USA 2013;110:12984–9. 23872845 10.1073/pnas.1304045110 PMC3740835

[4] Mashalidis EH, Śledź P, Lang S, Abell C A Three-stage biophysical screening cascade for fragment-based drug discovery. Nat Protoc 2013;8:2309–24. 24157549 10.1038/nprot.2013.130

[5] Bollag G, Hirth P, Tsai J, et al. Clinical efficacy of a RAF inhibitor needs broad target blockade in BRAF-mutant melanoma. Nature 2010;467:596–9. 20823850 10.1038/nature09454 PMC2948082

[6] Roberts A, Davids M, Pagel JM, et al. Targeting BCL2 with venetoclax in relapsed chronic lymphocytic leukemia. N Engl J Med 2016;374:311–22. 26639348 10.1056/NEJMoa1513257 PMC7107002

[7] Chen IJ, Hubbard RE Lessons for fragment library design: analysis of output from multiple screening campaigns. J Comput Aided Mol Des 2009;23:603–20. 19495994 10.1007/s10822-009-9280-5

[8] Harner MJ, Mueller L, Robbins KJ, Reily MD NMR in drug design. Arch Biochem Biophys 2017;628:132–47. 28619618 10.1016/j.abb.2017.06.005

[9] Mayer M, Meyer B Characterization of ligand binding by saturation transfer difference NMR spectroscopy. Angew Chem Int Ed Engl 1999;38:1784–8. 29711196 10.1002/(SICI)1521-3773(19990614)38:12<1784::AID-ANIE1784>3.0.CO;2-Q

[10] Meyer B, Peters T NMR spectroscopy techniques for screening and identifying ligand binding to protein receptors. Angew Chem Int Ed Engl 2003;42:864–90. 12596167 10.1002/anie.200390233

[11] Cala O, Guilliere F, Krimm I NMR-based analysis of protein-ligand interactions. Anal Bioanal Chem 2014;406:943–56. 23591643 10.1007/s00216-013-6931-0

[12] Wagstaff JL, Taylor SL, Howard MJ Recent developments and applications of saturation transfer difference nuclear magnetic resonance (STD NMR) spectroscopy. Mol Biosyst 2013;9:571–7. 23232937 10.1039/c2mb25395j

[13] Cala O, Krimm I Ligand-orientation based fragment selection in STD NMR screening. J Med Chem 2015;58:8739–42. 26492576 10.1021/acs.jmedchem.5b01114

[14] Dalvit C, Pevarello P, Tatò M, et al. Identification of compounds with binding affinity to proteins via magnetization transfer from bulk water. J Biomol NMR 2000;18:65–8. 11061229 10.1023/a:1008354229396

[15] Dalvit C, Fogliatto G, Stewart A, et al. WaterLOGSY as a method for primary NMR screening: practical aspects and range of applicability. J Biomol NMR 2001;21:349–59. 11824754 10.1023/a:1013302231549

[16] Huang R, Leung IKH Chapter fourteen - protein–small molecule interactions by WaterLOGSY In Joshua Wand A, editor. Methods in enzymology. Vol. 615 London: Academic Press; 2019:477–500. 10.1016/bs.mie.2018.08.020 30638539

[17] Huang R, Bonnichon A, Claridge TD, Leung IK Protein-ligand binding affinity determination by the waterLOGSY method: an optimised approach considering ligand rebinding. Sci Rep 2017;7:43727. 28256624 10.1038/srep43727 PMC5335602

[18] Antanasijevic A, Ramirez B, Caffrey M Comparison of the sensitivities of WaterLOGSY and saturation transfer difference NMR experiments. J Biomol NMR 2014;60:37–44. 25015532 10.1007/s10858-014-9848-9 PMC4201884

[19] Antanasijevic A, Kingsley C, Basu A, et al. Application of virus-like particles (VLP) to NMR characterization of viral membrane protein interactions. J Biomol NMR 2016;64:255–65. 26921030 10.1007/s10858-016-0025-1 PMC4826305

[20] Chu S, Zhou G, Gochin M Evaluation of ligand-based NMR screening methods to characterize small molecule binding to HIV-1 glycoprotein-41. Org Biomol Chem 2017;15:5210–9. 28590477 10.1039/c7ob00954b PMC5530879

[21] Chappuis Q, Milani J, Vuichoud B, et al. Hyperpolarized water to study protein–ligand interactions. J Phys Chem Lett 2015;6:1674–8. 26263332 10.1021/acs.jpclett.5b00403

[22] Johnson EC, Feher VA, Peng JW, et al. Application of NMR SHAPES screening to an RNA target. J Am Chem Soc 2003;125:15724–5. 14677945 10.1021/ja037499s

[23] Asencio-Hernández J, Kieffer B, Delsuc MA NMR WaterLOGSY reveals weak binding of bisphenol A with amyloid fibers of a conserved 11 residue peptide from androgen receptor. PLoS One 2016;11:e0161948. 27583469 10.1371/journal.pone.0161948 PMC5008648

[24] Ludwig C, Michiels PJA, Wu X, et al. SALMON: solvent accessibility, ligand binding, and mapping of ligand orientation by NMR spectroscopy. J Med Chem 2008;51:1–3. 18062662 10.1021/jm701020f

[25] Ludwig C, Michiels PJA, Lodi A, et al. Evaluation of solvent accessibility epitopes for different dehydrogenase inhibitors. ChemMedChem 2008;3:1371–6. 18576452 10.1002/cmdc.200800110

[26] Geist L, Mayer M, Cockcroft XL, et al. Direct NMR probing of hydration shells of protein ligand interfaces and its application to drug design. J Med Chem 2017;60:8708–15. 28910100 10.1021/acs.jmedchem.7b00845

[27] Aguirre C, ten Brink T, Guichou JF, et al. Comparing binding modes of analogous fragments using NMR in fragment-based drug design: application to PRDX5. PLoS One 2014;9:e102300. 25025339 10.1371/journal.pone.0102300 PMC4099364

[28] Barelier S, Pons J, Gehring K, et al. Ligand specificity in fragment-based drug design. J Med Chem 2010;53:5256–66. 20575554 10.1021/jm100496j

[29] Trott O, Olson AJ AutoDock Vina: improving the speed and accuracy of docking with a new scoring function, efficient optimization, and multithreading. J Comput Chem 2010;31:455–61. 19499576 10.1002/jcc.21334 PMC3041641

[30] Mayer M, Meyer B Group epitope mapping by saturation transfer difference NMR to identify segments of a ligand in direct contact with a protein receptor. J Am Chem Soc 2001;123:6108–17. 11414845 10.1021/ja0100120

[31] Fasano M, Curry S, Terreno E, et al. The extraordinary ligand binding properties of human serum albumin. IUBMB Life 2005;57:787–96. 16393781 10.1080/15216540500404093

[32] Murray CW, Carr MG, Callaghan O, et al. Fragment-based drug discovery applied to Hsp90. Discovery of two lead series with high ligand efficiency. J Med Chem 2010;53:5942–55. 20718493 10.1021/jm100059d

[33] Petros AM, Dinges J, Augeri DJ, et al. Discovery of a potent inhibitor of the antiapoptotic protein Bcl-xL from NMR and parallel synthesis. J Med Chem 2006;49:656–63. 16420051 10.1021/jm0507532

[34] Szczepina MG, Bleile D, Müllegger J, et al. WaterLOGSY NMR experiments in conjunction with molecular-dynamics simulations identify immobilized water molecules that bridge peptide mimic MDWNMHAA to anticarbohydrate antibody SYA/J6. Chem Eur J 2011;17:11438–45. 21887835 10.1002/chem.201101464

