# Supplementary material for: 1D NMR WaterLOGSY as an efficient method for fragment-based lead discovery
Source: J Enzyme Inhib Med Chem. 2019 Jul 9;34(1):1218–25. doi: 10.1080/14756366.2019.1636235 (PMC6691826; doi:10.1080/14756366.2019.1636235)
Supplement: Supplemental Material [file IENZ_A_1636235_SM0471.docx]

Table S1. Variation of WaterLOGSY intensities with mixing time.

| **Protein** | **Compound** | **Relative intensities of WaterLOGSY at 0.25 s, 0.75 s and 1.5 s mixing times** |
| --- | --- | --- |
| GP | Benzoyleneurea  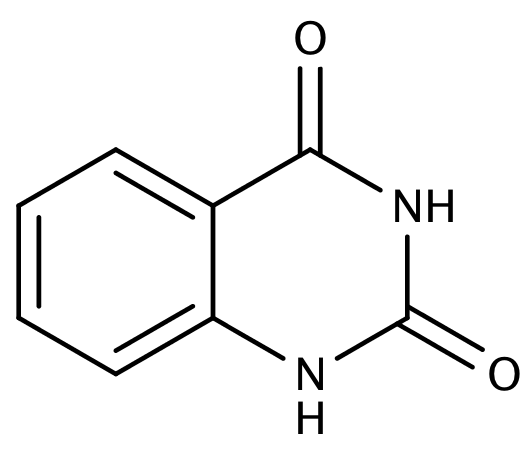  * | * signal increases at low mixing time |
| GP | Benzimidazole  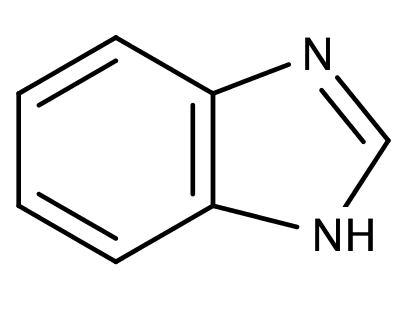  * | * signal increases at low mixing time |
| GP | 3,4-Dihydroquinazolin-4-one  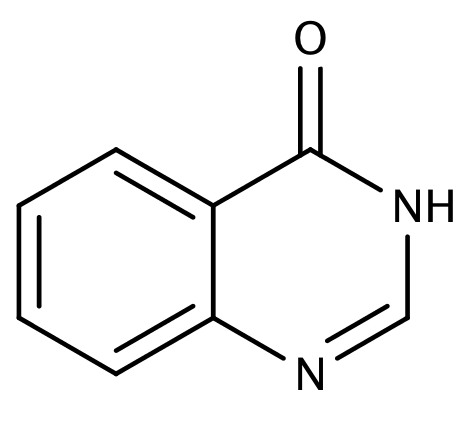  * | * signal increases at low mixing time |
| GP | 7-Azaindole  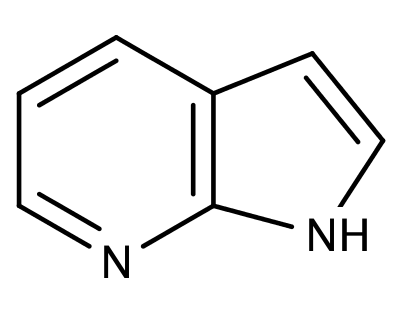  *  * | * signals increase at low mixing time |
| GP | 3-Aminobenzonitrile  *  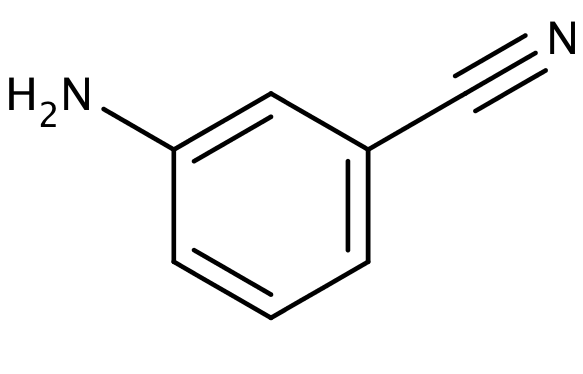 | * signal increases at low mixing time |
| GP | Chromone  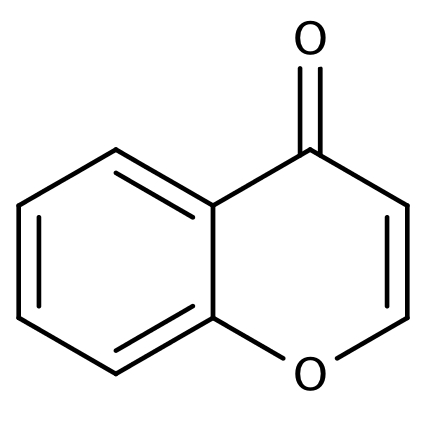 | no significant variation |
| GP | 1,2,3,4-Tetrahydro-6 quinolinecarboxylic acid  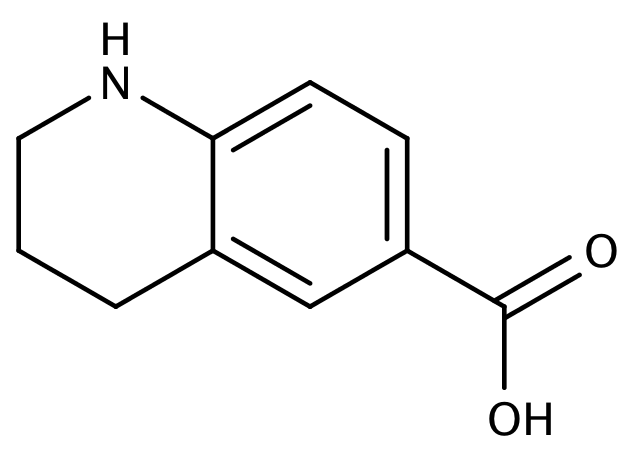 | no significant variation (aromatic protons) |
| GP | 2-Phenylphenol  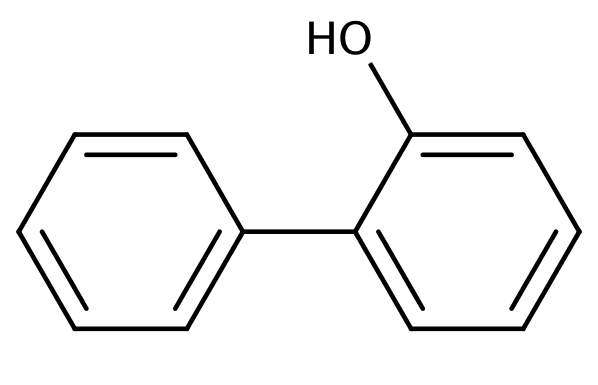  * | * signal increases at low mixing time |
| GP | 4-Phenoxybenzoic acid  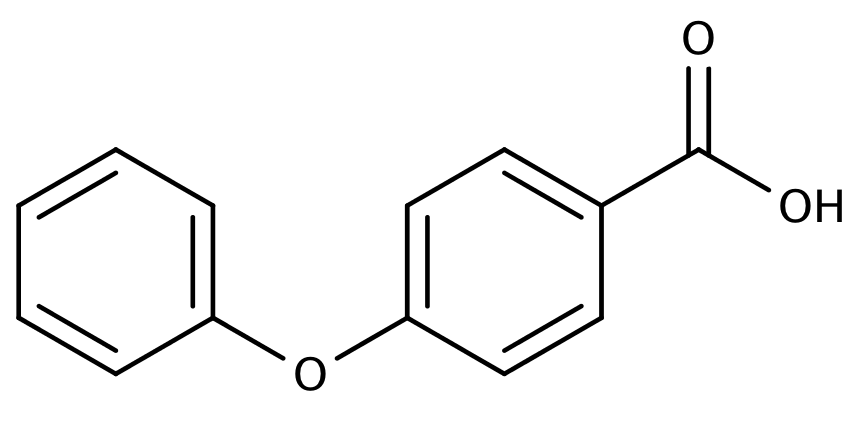 | no significant variation |

Table S1. Variation of WaterLOGSY intensities with mixing time (Table continued from previous page).

| **Protein** | **Compound** | **Relative intensities of WaterLOGSY at 0.25 s, 0.75 s and 1.5 s mixing times** |
| --- | --- | --- |
| HSA | 4-Methylcatechol  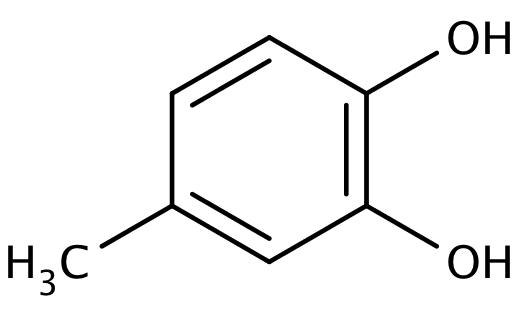 | * signals increase at low mixing time |
| HSA | Benzoyleneurea  *  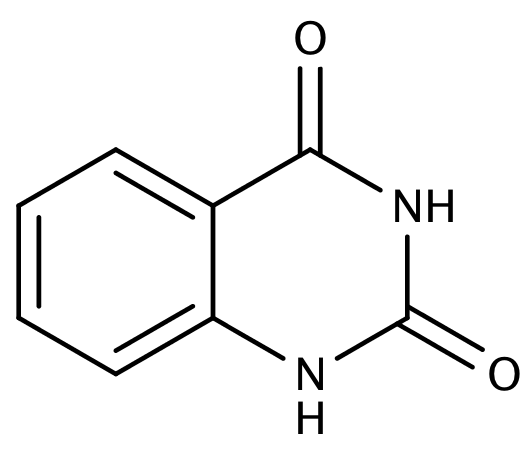  * | * signals increase at low mixing time |
| HSA | Benzimidazole  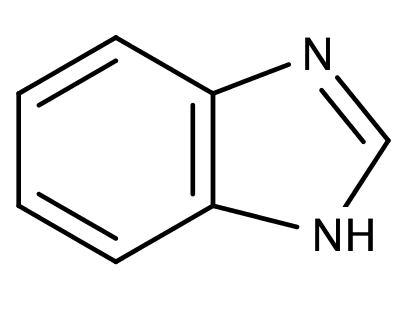  * | * signal increases at low mixing time |
| HSA | 3,4-Dihydroquinazolin-4-one  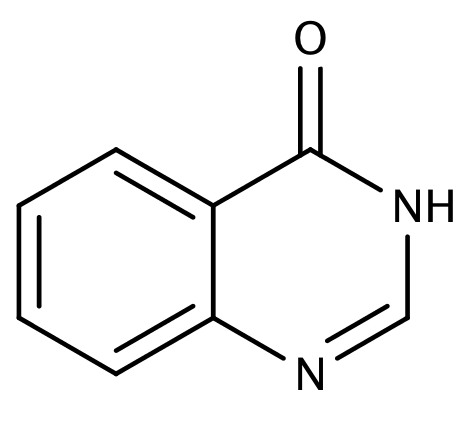  * | * signal increases at low mixing time |
| HSA | 3-Aminobenzonitrile  *  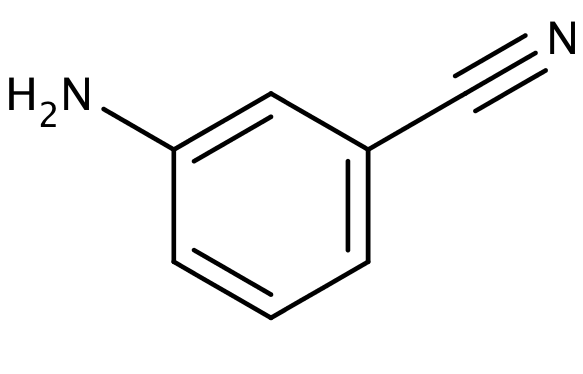  * | * signals increase at low mixing time |
| HSA | Indole  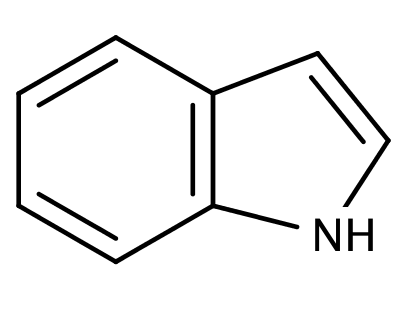  *  * | * signals increase at low mixing time |
| HSA | 5-Chloroindole  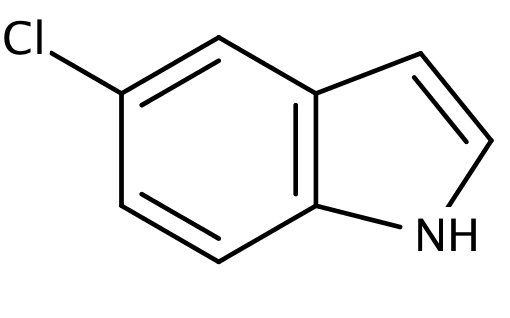  *  * | * signals increase at low mixing time |
| HSA | L-Tryptophane  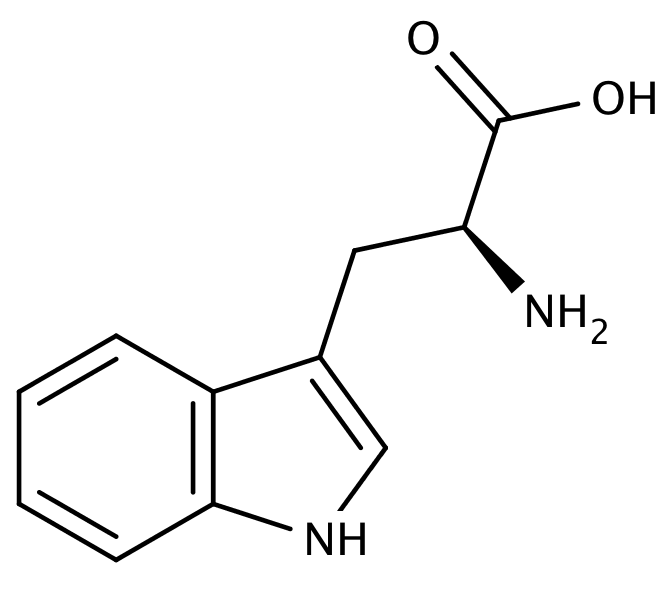  * | * signal increases at low mixing time (aromatic protons) |

Table S1. Variation of WaterLOGSY intensities with mixing time (Table continued from previous page).

| **Protein** | **Compound** | **Relative intensities of WaterLOGSY at 0.25 s, 0.75 s and 1.5 s mixing times** |
| --- | --- | --- |
| PRDX5 | 4-Methylcatechol  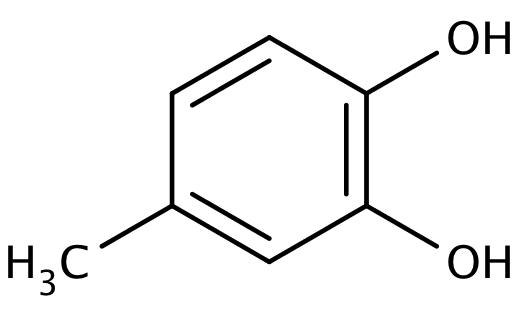  * | * signal increases at low mixing time |
| PRDX5 | 4-tert-Butylcatechol  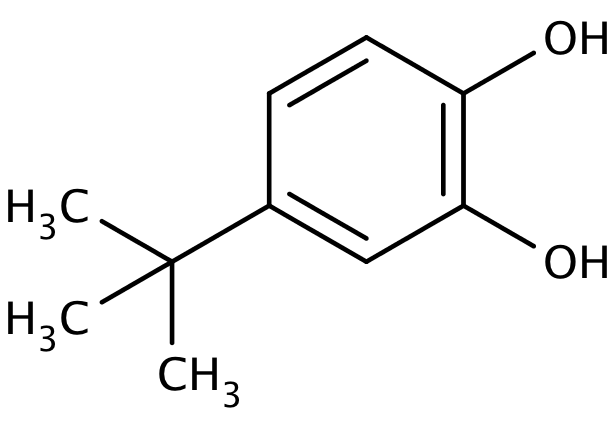  * | * signal increases at low mixing time |
| PRDX5 | [1,1'-Biphenyl]-3,4-diol  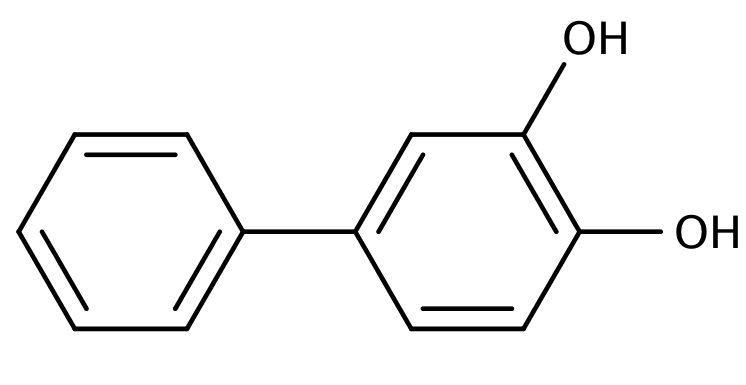  *  * | * signals increase at low mixing time |
